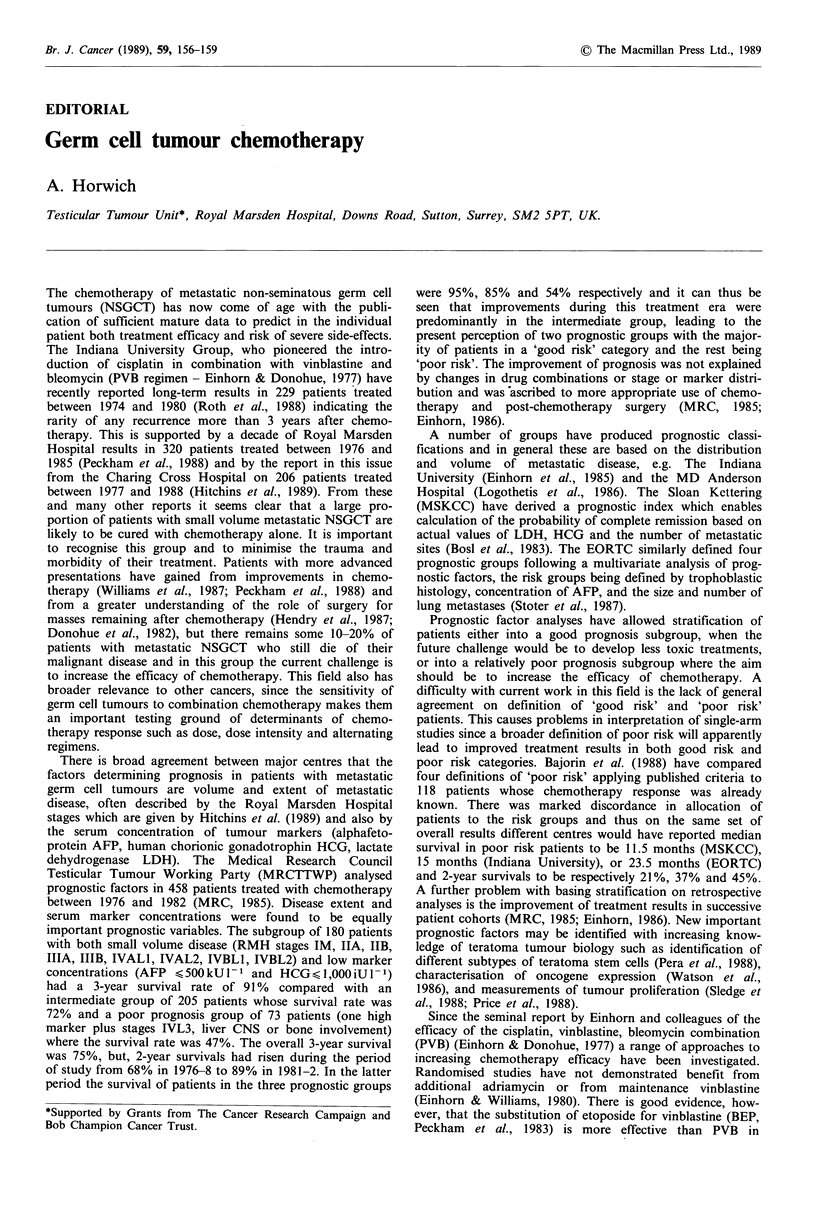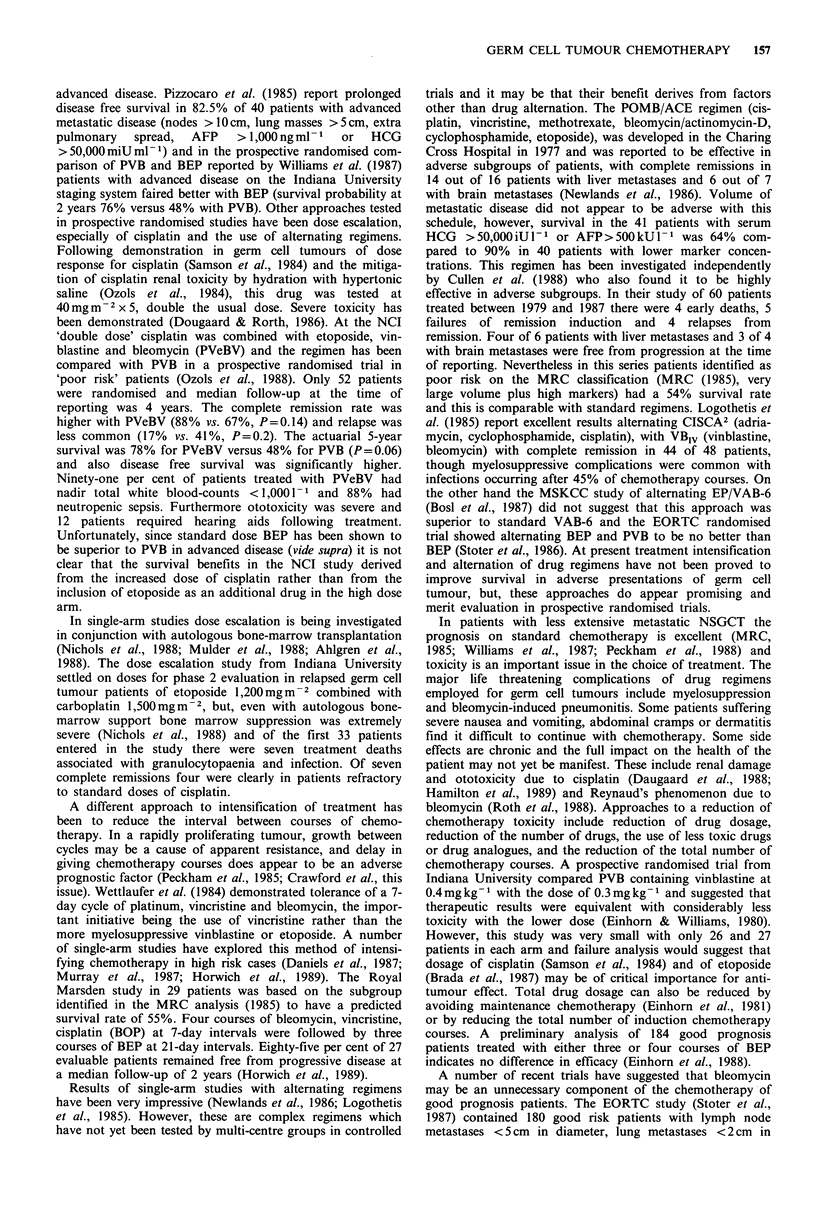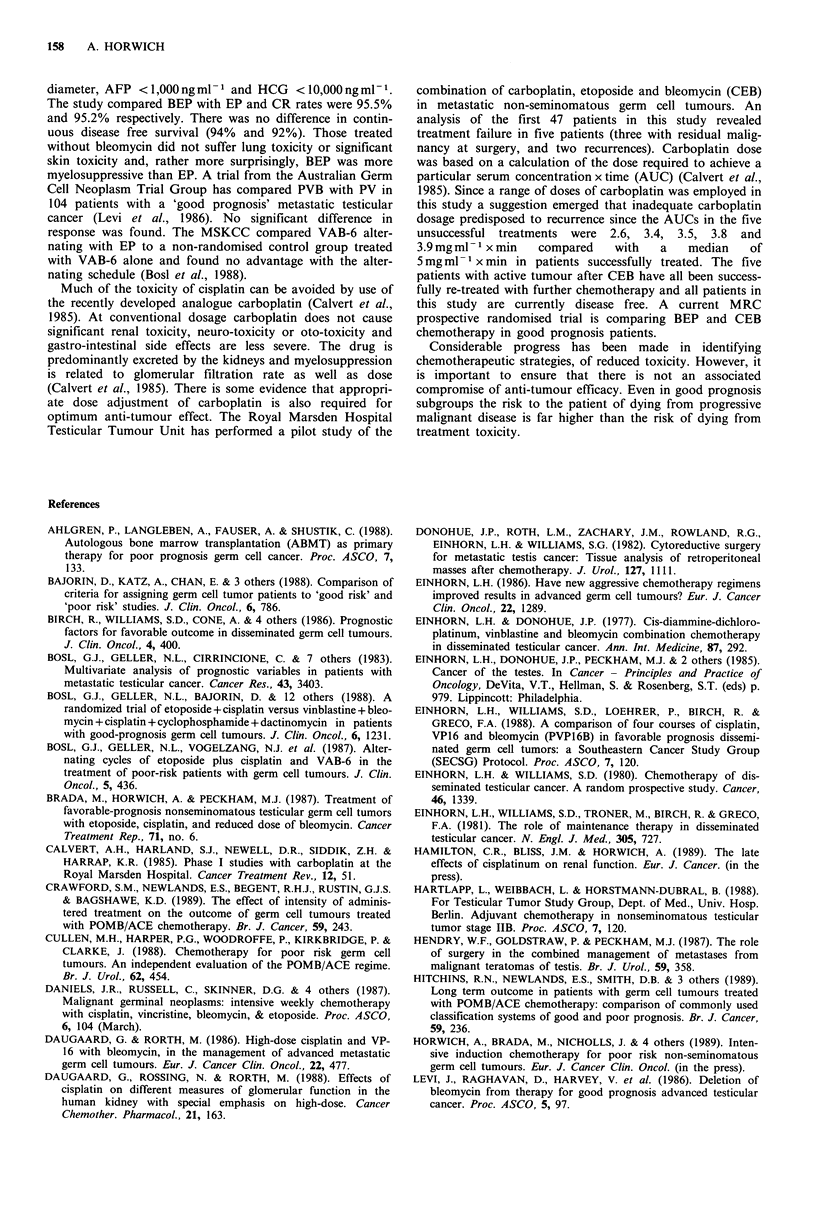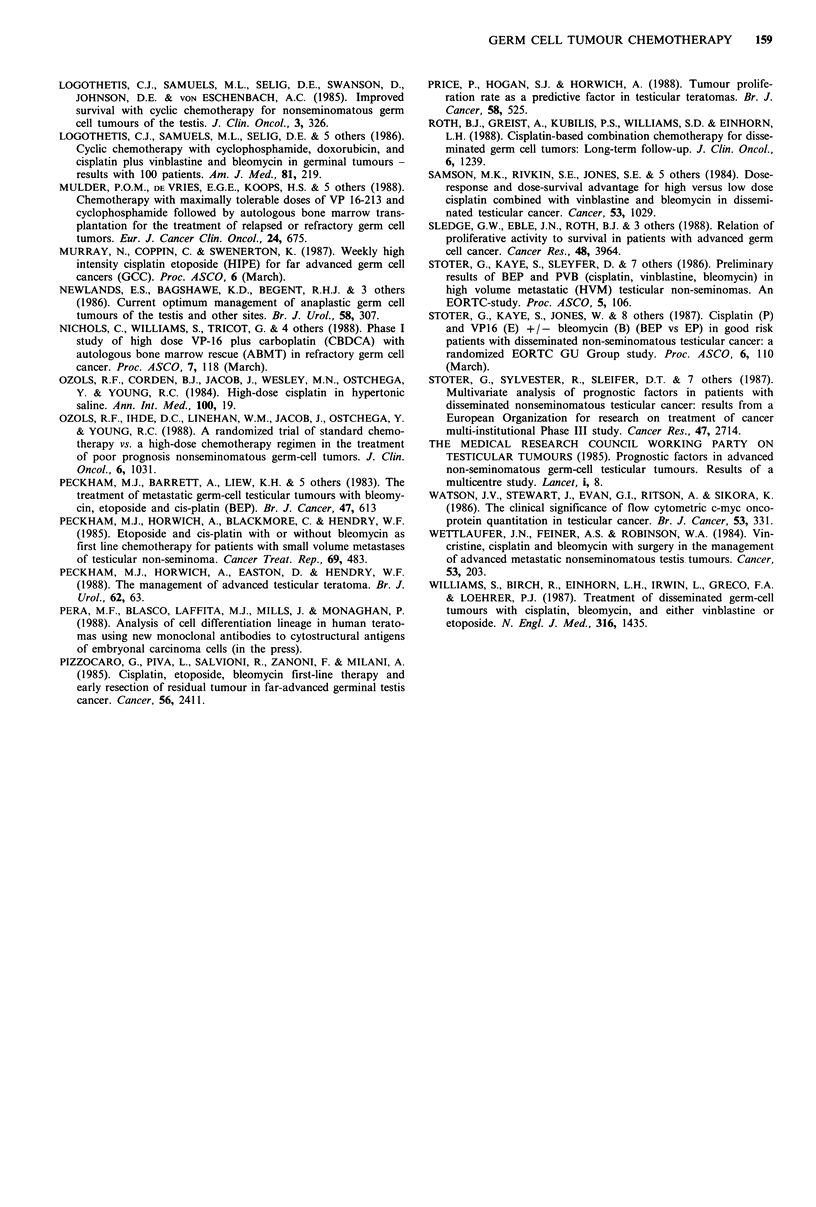# Germ cell tumour chemotherapy.

**DOI:** 10.1038/bjc.1989.33

**Published:** 1989-02

**Authors:** A. Horwich

**Affiliations:** Testicular Tumour Unit, Royal Marsden Hospital, Sutton, Surrey, UK.


*Br. J. Cancer* (1989), **59**, 156–159 © The Macmillan Press Ltd., 1989

EDITORIAL

### Germ cell tumour chemotherapy

A. Horwich

*Testicular Tumour Unit*, Royal Marsden Hospital, Downs Road, Sutton, Surrey, SM2 5PT, UK.*

The chemotherapy of metastatic non-seminatous germ cell tumours (NSGCT) has now come of age with the publication of sufficient mature data to predict in the individual patient both treatment efficacy and risk of severe side-effects. The Indiana University Group, who pioneered the introduction of cisplatin in combination with vinblastine and bleomycin (PVB regimen – Einhorn & Donohue, 1977) have recently reported long-term results in 229 patients treated between 1974 and 1980 (Roth *et al.*, 1988) indicating the rarity of any recurrence more than 3 years after chemotherapy. This is supported by a decade of Royal Marsden Hospital results in 320 patients treated between 1976 and 1985 (Peckham *et al.*, 1988) and by the report in this issue from the Charing Cross Hospital on 206 patients treated between 1977 and 1988 (Hitchins *et al.*, 1989). From these and many other reports it seems clear that a large proportion of patients with small volume metastatic NSGCT are likely to be cured with chemotherapy alone. It is important to recognise this group and to minimise the trauma and morbidity of their treatment. Patients with more advanced presentations have gained from improvements in chemotherapy (Williams *et al.*, 1987; Peckham *et al.*, 1988) and from a greater understanding of the role of surgery for masses remaining after chemotherapy (Hendry *et al.*, 1987; Donohue *et al.*, 1982), but there remains some 10–20% of patients with metastatic NSGCT who still die of their malignant disease and in this group the current challenge is to increase the efficacy of chemotherapy. This field also has broader relevance to other cancers, since the sensitivity of germ cell tumours to combination chemotherapy makes them an important testing ground of determinants of chemotherapy response such as dose, dose intensity and alternating regimens.

There is broad agreement between major centres that the factors determining prognosis in patients with metastatic germ cell tumours are volume and extent of metastatic disease, often described by the Royal Marsden Hospital stages which are given by Hitchins *et al.* (1989) and also by the serum concentration of tumour markers (alphafetoprotein AFP, human chorionic gonadotrophin HCG, lactate dehydrogenase LDH). The Medical Research Council Testicular Tumour Working Party (MRCTTWP) analysed prognostic factors in 458 patients treated with chemotherapy between 1976 and 1982 (MRC, 1985). Disease extent and serum marker concentrations were found to be equally important prognostic variables. The subgroup of 180 patients with both small volume disease (RMH stages IM, IIA, IIB, IIIA, IIIB, IVAL1, IVAL2, IVBL1, IVBL2) and low marker concentrations (AFP $\leqslant 500\,kU\,l^{-1}$ and HCG $\leqslant 1{,}000\,iU\,l^{-1}$) had a 3-year survival rate of 91% compared with an intermediate group of 205 patients whose survival rate was 72% and a poor prognosis group of 73 patients (one high marker plus stages IVL3, liver CNS or bone involvement) where the survival rate was 47%. The overall 3-year survival was 75%, but, 2-year survivals had risen during the period of study from 68% in 1976–8 to 89% in 1981–2. In the latter period the survival of patients in the three prognostic groups were 95%, 85% and 54% respectively and it can thus be seen that improvements during this treatment era were predominantly in the intermediate group, leading to the present perception of two prognostic groups with the majority of patients in a 'good risk' category and the rest being 'poor risk'. The improvement of prognosis was not explained by changes in drug combinations or stage or marker distribution and was ascribed to more appropriate use of chemotherapy and post-chemotherapy surgery (MRC, 1985; Einhorn, 1986).

A number of groups have produced prognostic classifications and in general these are based on the distribution and volume of metastatic disease, e.g. The Indiana University (Einhorn *et al.*, 1985) and the MD Anderson Hospital (Logothetis *et al.*, 1986). The Sloan Kettering (MSKCC) have derived a prognostic index which enables calculation of the probability of complete remission based on actual values of LDH, HCG and the number of metastatic sites (Bosl *et al.*, 1983). The EORTC similarly defined four prognostic groups following a multivariate analysis of prognostic factors, the risk groups being defined by trophoblastic histology, concentration of AFP, and the size and number of lung metastases (Stoter *et al.*, 1987).

Prognostic factor analyses have allowed stratification of patients either into a good prognosis subgroup, when the future challenge would be to develop less toxic treatments, or into a relatively poor prognosis subgroup where the aim should be to increase the efficacy of chemotherapy. A difficulty with current work in this field is the lack of general agreement on definition of 'good risk' and 'poor risk' patients. This causes problems in interpretation of single-arm studies since a broader definition of poor risk will apparently lead to improved treatment results in both good risk and poor risk categories. Bajorin *et al.* (1988) have compared four definitions of 'poor risk' applying published criteria to 118 patients whose chemotherapy response was already known. There was marked discordance in allocation of patients to the risk groups and thus on the same set of overall results different centres would have reported median survival in poor risk patients to be 11.5 months (MSKCC), 15 months (Indiana University), or 23.5 months (EORTC) and 2-year survivals to be respectively 21%, 37% and 45%. A further problem with basing stratification on retrospective analyses is the improvement of treatment results in successive patient cohorts (MRC, 1985; Einhorn, 1986). New important prognostic factors may be identified with increasing knowledge of teratoma tumour biology such as identification of different subtypes of teratoma stem cells (Pera *et al.*, 1988), characterisation of oncogene expression (Watson *et al.*, 1986), and measurements of tumour proliferation (Sledge *et al.*, 1988; Price *et al.*, 1988).

Since the seminal report by Einhorn and colleagues of the efficacy of the cisplatin, vinblastine, bleomycin combination (PVB) (Einhorn & Donohue, 1977) a range of approaches to increasing chemotherapy efficacy have been investigated. Randomised studies have not demonstrated benefit from additional adriamycin or from maintenance vinblastine (Einhorn & Williams, 1980). There is good evidence, however, that the substitution of etoposide for vinblastine (BEP, Peckham *et al.*, 1983) is more effective than PVB in

*Supported by Grants from The Cancer Research Campaign and Bob Champion Cancer Trust.

advanced disease. Pizzocaro *et al.* (1985) report prolonged disease free survival in 82.5% of 40 patients with advanced metastatic disease (nodes >10 cm, lung masses >5 cm, extra pulmonary spread, AFP $>1,000\,\mathrm{ng\,ml^{-1}}$ or HCG $>50,000\,\mathrm{miU\,ml^{-1}}$) and in the prospective randomised comparison of PVB and BEP reported by Williams *et al.* (1987) patients with advanced disease on the Indiana University staging system faired better with BEP (survival probability at 2 years 76% versus 48% with PVB). Other approaches tested in prospective randomised studies have been dose escalation, especially of cisplatin and the use of alternating regimens. Following demonstration in germ cell tumours of dose response for cisplatin (Samson *et al.*, 1984) and the mitigation of cisplatin renal toxicity by hydration with hypertonic saline (Ozols *et al.*, 1984), this drug was tested at $40\,\mathrm{mg\,m^{-2}} \times 5$, double the usual dose. Severe toxicity has been demonstrated (Dougaard & Rorth, 1986). At the NCI 'double dose' cisplatin was combined with etoposide, vinblastine and bleomycin (PVeBV) and the regimen has been compared with PVB in a prospective randomised trial in 'poor risk' patients (Ozols *et al.*, 1988). Only 52 patients were randomised and median follow-up at the time of reporting was 4 years. The complete remission rate was higher with PVeBV (88% *vs.* 67%, $P=0.14$) and relapse was less common (17% *vs.* 41%, $P=0.2$). The actuarial 5-year survival was 78% for PVeBV versus 48% for PVB ($P=0.06$) and also disease free survival was significantly higher. Ninety-one per cent of patients treated with PVeBV had nadir total white blood-counts $<1,000\,\mathrm{l^{-1}}$ and 88% had neutropenic sepsis. Furthermore ototoxicity was severe and 12 patients required hearing aids following treatment. Unfortunately, since standard dose BEP has been shown to be superior to PVB in advanced disease (*vide supra*) it is not clear that the survival benefits in the NCI study derived from the increased dose of cisplatin rather than from the inclusion of etoposide as an additional drug in the high dose arm.

In single-arm studies dose escalation is being investigated in conjunction with autologous bone-marrow transplantation (Nichols *et al.*, 1988; Mulder *et al.*, 1988; Ahlgren *et al.*, 1988). The dose escalation study from Indiana University settled on doses for phase 2 evaluation in relapsed germ cell tumour patients of etoposide $1,200\,\mathrm{mg\,m^{-2}}$ combined with carboplatin $1,500\,\mathrm{mg\,m^{-2}}$, but, even with autologous bone-marrow support bone marrow suppression was extremely severe (Nichols *et al.*, 1988) and of the first 33 patients entered in the study there were seven treatment deaths associated with granulocytopaenia and infection. Of seven complete remissions four were clearly in patients refractory to standard doses of cisplatin.

A different approach to intensification of treatment has been to reduce the interval between courses of chemotherapy. In a rapidly proliferating tumour, growth between cycles may be a cause of apparent resistance, and delay in giving chemotherapy courses does appear to be an adverse prognostic factor (Peckham *et al.*, 1985; Crawford *et al.*, this issue). Wettlaufer *et al.* (1984) demonstrated tolerance of a 7-day cycle of platinum, vincristine and bleomycin, the important initiative being the use of vincristine rather than the more myelosuppressive vinblastine or etoposide. A number of single-arm studies have explored this method of intensifying chemotherapy in high risk cases (Daniels *et al.*, 1987; Murray *et al.*, 1987; Horwich *et al.*, 1989). The Royal Marsden study in 29 patients was based on the subgroup identified in the MRC analysis (1985) to have a predicted survival rate of 55%. Four courses of bleomycin, vincristine, cisplatin (BOP) at 7-day intervals were followed by three courses of BEP at 21-day intervals. Eighty-five per cent of 27 evaluable patients remained free from progressive disease at a median follow-up of 2 years (Horwich *et al.*, 1989).

Results of single-arm studies with alternating regimens have been very impressive (Newlands *et al.*, 1986; Logothetis *et al.*, 1985). However, these are complex regimens which have not yet been tested by multi-centre groups in controlled trials and it may be that their benefit derives from factors other than drug alternation. The POMB/ACE regimen (cisplatin, vincristine, methotrexate, bleomycin/actinomycin-D, cyclophosphamide, etoposide), was developed in the Charing Cross Hospital in 1977 and was reported to be effective in adverse subgroups of patients, with complete remissions in 14 out of 16 patients with liver metastases and 6 out of 7 with brain metastases (Newlands *et al.*, 1986). Volume of metastatic disease did not appear to be adverse with this schedule, however, survival in the 41 patients with serum HCG $>50,000\,\mathrm{iU\,l^{-1}}$ or AFP$>500\,\mathrm{kU\,l^{-1}}$ was 64% compared to 90% in 40 patients with lower marker concentrations. This regimen has been investigated independently by Cullen *et al.* (1988) who also found it to be highly effective in adverse subgroups. In their study of 60 patients treated between 1979 and 1987 there were 4 early deaths, 5 failures of remission induction and 4 relapses from remission. Four of 6 patients with liver metastases and 3 of 4 with brain metastases were free from progression at the time of reporting. Nevertheless in this series patients identified as poor risk on the MRC classification (MRC (1985), very large volume plus high markers) had a 54% survival rate and this is comparable with standard regimens. Logothetis *et al.* (1985) report excellent results alternating $\mathrm{CISCA^2}$ (adriamycin, cyclophosphamide, cisplatin), with $\mathrm{VB_{IV}}$ (vinblastine, bleomycin) with complete remission in 44 of 48 patients, though myelosuppressive complications were common with infections occurring after 45% of chemotherapy courses. On the other hand the MSKCC study of alternating EP/VAB-6 (Bosl *et al.*, 1987) did not suggest that this approach was superior to standard VAB-6 and the EORTC randomised trial showed alternating BEP and PVB to be no better than BEP (Stoter *et al.*, 1986). At present treatment intensification and alternation of drug regimens have not been proved to improve survival in adverse presentations of germ cell tumour, but, these approaches do appear promising and merit evaluation in prospective randomised trials.

In patients with less extensive metastatic NSGCT the prognosis on standard chemotherapy is excellent (MRC, 1985; Williams *et al.*, 1987; Peckham *et al.*, 1988) and toxicity is an important issue in the choice of treatment. The major life threatening complications of drug regimens employed for germ cell tumours include myelosuppression and bleomycin-induced pneumonitis. Some patients suffering severe nausea and vomiting, abdominal cramps or dermatitis find it difficult to continue with chemotherapy. Some side effects are chronic and the full impact on the health of the patient may not yet be manifest. These include renal damage and ototoxicity due to cisplatin (Daugaard *et al.*, 1988; Hamilton *et al.*, 1989) and Reynaud's phenomenon due to bleomycin (Roth *et al.*, 1988). Approaches to a reduction of chemotherapy toxicity include reduction of drug dosage, reduction of the number of drugs, the use of less toxic drugs or drug analogues, and the reduction of the total number of chemotherapy courses. A prospective randomised trial from Indiana University compared PVB containing vinblastine at $0.4\,\mathrm{mg\,kg^{-1}}$ with the dose of $0.3\,\mathrm{mg\,kg^{-1}}$ and suggested that therapeutic results were equivalent with considerably less toxicity with the lower dose (Einhorn & Williams, 1980). However, this study was very small with only 26 and 27 patients in each arm and failure analysis would suggest that dosage of cisplatin (Samson *et al.*, 1984) and of etoposide (Brada *et al.*, 1987) may be of critical importance for anti-tumour effect. Total drug dosage can also be reduced by avoiding maintenance chemotherapy (Einhorn *et al.*, 1981) or by reducing the total number of induction chemotherapy courses. A preliminary analysis of 184 good prognosis patients treated with either three or four courses of BEP indicates no difference in efficacy (Einhorn *et al.*, 1988).

A number of recent trials have suggested that bleomycin may be an unnecessary component of the chemotherapy of good prognosis patients. The EORTC study (Stoter *et al.*, 1987) contained 180 good risk patients with lymph node metastases <5 cm in diameter, lung metastases <2 cm in diameter, AFP $<1,000\,ng\,ml^{-1}$ and HCG $<10,000\,ng\,ml^{-1}$. The study compared BEP with EP and CR rates were 95.5% and 95.2% respectively. There was no difference in continuous disease free survival (94% and 92%). Those treated without bleomycin did not suffer lung toxicity or significant skin toxicity and, rather more surprisingly, BEP was more myelosuppressive than EP. A trial from the Australian Germ Cell Neoplasm Trial Group has compared PVB with PV in 104 patients with a 'good prognosis' metastatic testicular cancer (Levi *et al.*, 1986). No significant difference in response was found. The MSKCC compared VAB-6 alternating with EP to a non-randomised control group treated with VAB-6 alone and found no advantage with the alternating schedule (Bosl *et al.*, 1988).

Much of the toxicity of cisplatin can be avoided by use of the recently developed analogue carboplatin (Calvert *et al.*, 1985). At conventional dosage carboplatin does not cause significant renal toxicity, neuro-toxicity or oto-toxicity and gastro-intestinal side effects are less severe. The drug is predominantly excreted by the kidneys and myelosuppression is related to glomerular filtration rate as well as dose (Calvert *et al.*, 1985). There is some evidence that appropriate dose adjustment of carboplatin is also required for optimum anti-tumour effect. The Royal Marsden Hospital Testicular Tumour Unit has performed a pilot study of the combination of carboplatin, etoposide and bleomycin (CEB) in metastatic non-seminomatous germ cell tumours. An analysis of the first 47 patients in this study revealed treatment failure in five patients (three with residual malignancy at surgery, and two recurrences). Carboplatin dose was based on a calculation of the dose required to achieve a particular serum concentration × time (AUC) (Calvert *et al.*, 1985). Since a range of doses of carboplatin was employed in this study a suggestion emerged that inadequate carboplatin dosage predisposed to recurrence since the AUCs in the five unsuccessful treatments were 2.6, 3.4, 3.5, 3.8 and $3.9\,mg\,ml^{-1} \times min$ compared with a median of $5\,mg\,ml^{-1} \times min$ in patients successfully treated. The five patients with active tumour after CEB have all been successfully re-treated with further chemotherapy and all patients in this study are currently disease free. A current MRC prospective randomised trial is comparing BEP and CEB chemotherapy in good prognosis patients.

Considerable progress has been made in identifying chemotherapeutic strategies, of reduced toxicity. However, it is important to ensure that there is not an associated compromise of anti-tumour efficacy. Even in good prognosis subgroups the risk to the patient of dying from progressive malignant disease is far higher than the risk of dying from treatment toxicity.

### References

AHLGREN, P., LANGLEBEN, A., FAUSER, A. & SHUSTIK, C. (1988). Autologous bone marrow transplantation (ABMT) as primary therapy for poor prognosis germ cell cancer. *Proc. ASCO*, **7**, 133.

BAJORIN, D., KATZ, A., CHAN, E. & 3 others (1988). Comparison of criteria for assigning germ cell tumor patients to 'good risk' and 'poor risk' studies. *J. Clin. Oncol.*, **6**, 786.

BIRCH, R., WILLIAMS, S.D., CONE, A. & 4 others (1986). Prognostic factors for favorable outcome in disseminated germ cell tumours. *J. Clin. Oncol.*, **4**, 400.

BOSL, G.J., GELLER, N.L., CIRRINCIONE, C. & 7 others (1983). Multivariate analysis of prognostic variables in patients with metastatic testicular cancer. *Cancer Res.*, **43**, 3403.

BOSL, G.J., GELLER, N.L., BAJORIN, D. & 12 others (1988). A randomized trial of etoposide + cisplatin versus vinblastine + bleomycin + cisplatin + cyclophosphamide + dactinomycin in patients with good-prognosis germ cell tumours. *J. Clin. Oncol.*, **6**, 1231.

BOSL, G.J., GELLER, N.L., VOGELZANG, N.J. *et al.* (1987). Alternating cycles of etoposide plus cisplatin and VAB-6 in the treatment of poor-risk patients with germ cell tumours. *J. Clin. Oncol.*, **5**, 436.

BRADA, M., HORWICH, A. & PECKHAM, M.J. (1987). Treatment of favorable-prognosis nonseminomatous testicular germ cell tumors with etoposide, cisplatin, and reduced dose of bleomycin. *Cancer Treatment Rep.*, **71**, no. 6.

CALVERT, A.H., HARLAND, S.J., NEWELL, D.R., SIDDIK, Z.H. & HARRAP, K.R. (1985). Phase I studies with carboplatin at the Royal Marsden Hospital. *Cancer Treatment Rev.*, **12**, 51.

CRAWFORD, S.M., NEWLANDS, E.S., BEGENT, R.H.J., RUSTIN, G.J.S. & BAGSHAWE, K.D. (1989). The effect of intensity of administered treatment on the outcome of germ cell tumours treated with POMB/ACE chemotherapy. *Br. J. Cancer*, **59**, 243.

CULLEN, M.H., HARPER, P.G., WOODROFFE, P., KIRKBRIDGE, P. & CLARKE, J. (1988). Chemotherapy for poor risk germ cell tumours. An independent evaluation of the POMB/ACE regime. *Br. J. Urol.*, **62**, 454.

DANIELS, J.R., RUSSELL, C., SKINNER, D.G. & 4 others (1987). Malignant germinal neoplasms: intensive weekly chemotherapy with cisplatin, vincristine, bleomycin, & etoposide. *Proc. ASCO*, **6**, 104 (March).

DAUGAARD, G. & RORTH, M. (1986). High-dose cisplatin and VP-16 with bleomycin, in the management of advanced metastatic germ cell tumours. *Eur. J. Cancer Clin. Oncol.*, **22**, 477.

DAUGAARD, G., ROSSING, N. & RORTH, M. (1988). Effects of cisplatin on different measures of glomerular function in the human kidney with special emphasis on high-dose. *Cancer Chemother. Pharmacol.*, **21**, 163.

DONOHUE, J.P., ROTH, L.M., ZACHARY, J.M., ROWLAND, R.G., EINHORN, L.H. & WILLIAMS, S.G. (1982). Cytoreductive surgery for metastatic testis cancer: Tissue analysis of retroperitoneal masses after chemotherapy. *J. Urol.*, **127**, 1111.

EINHORN, L.H. (1986). Have new aggressive chemotherapy regimens improved results in advanced germ cell tumours? *Eur. J. Cancer Clin. Oncol.*, **22**, 1289.

EINHORN, L.H. & DONOHUE, J.P. (1977). Cis-diammine-dichloroplatinum, vinblastine and bleomycin combination chemotherapy in disseminated testicular cancer. *Ann. Int. Medicine*, **87**, 292.

EINHORN, L.H., DONOHUE, J.P., PECKHAM, M.J. & 2 others (1985). Cancer of the testes. In *Cancer – Principles and Practice of Oncology*, DeVita, V.T., Hellman, S. & Rosenberg, S.T. (eds) p. 979. Lippincott: Philadelphia.

EINHORN, L.H., WILLIAMS, S.D., LOEHRER, P., BIRCH, R. & GRECO, F.A. (1988). A comparison of four courses of cisplatin, VP16 and bleomycin (PVP16B) in favorable prognosis disseminated germ cell tumors: a Southeastern Cancer Study Group (SECSG) Protocol. *Proc. ASCO*, **7**, 120.

EINHORN, L.H. & WILLIAMS, S.D. (1980). Chemotherapy of disseminated testicular cancer. A random prospective study. *Cancer*, **46**, 1339.

EINHORN, L.H., WILLIAMS, S.D., TRONER, M., BIRCH, R. & GRECO, F.A. (1981). The role of maintenance therapy in disseminated testicular cancer. *N. Engl. J. Med.*, **305**, 727.

HAMILTON, C.R., BLISS, J.M. & HORWICH, A. (1989). The late effects of cisplatinum on renal function. *Eur. J. Cancer.* (in the press).

HARTLAPP, L., WEIBBACH, L. & HORSTMANN-DUBRAL, B. (1988). For Testicular Tumor Study Group, Dept. of Med., Univ. Hosp. Berlin. Adjuvant chemotherapy in nonseminomatous testicular tumor stage IIB. *Proc. ASCO*, **7**, 120.

HENDRY, W.F., GOLDSTRAW, P. & PECKHAM, M.J. (1987). The role of surgery in the combined management of metastases from malignant teratomas of testis. *Br. J. Urol.*, **59**, 358.

HITCHINS, R.N., NEWLANDS, E.S., SMITH, D.B. & 3 others (1989). Long term outcome in patients with germ cell tumours treated with POMB/ACE chemotherapy: comparison of commonly used classification systems of good and poor prognosis. *Br. J. Cancer*, **59**, 236.

HORWICH, A., BRADA, M., NICHOLLS, J. & 4 others (1989). Intensive induction chemotherapy for poor risk non-seminomatous germ cell tumours. *Eur. J. Cancer Clin. Oncol.* (in the press).

LEVI, J., RAGHAVAN, D., HARVEY, V. *et al.* (1986). Deletion of bleomycin from therapy for good prognosis advanced testicular cancer. *Proc. ASCO*, **5**, 97.

LOGOTHETIS, C.J., SAMUELS, M.L., SELIG, D.E., SWANSON, D., JOHNSON, D.E. & VON ESCHENBACH, A.C. (1985). Improved survival with cyclic chemotherapy for nonseminomatous germ cell tumours of the testis. *J. Clin. Oncol.*, **3**, 326.

LOGOTHETIS, C.J., SAMUELS, M.L., SELIG, D.E. & 5 others (1986). Cyclic chemotherapy with cyclophosphamide, doxorubicin, and cisplatin plus vinblastine and bleomycin in germinal tumours – results with 100 patients. *Am. J. Med.*, **81**, 219.

MULDER, P.O.M., DE VRIES, E.G.E., KOOPS, H.S. & 5 others (1988). Chemotherapy with maximally tolerable doses of VP 16-213 and cyclophosphamide followed by autologous bone marrow transplantation for the treatment of relapsed or refractory germ cell tumors. *Eur. J. Cancer Clin. Oncol.*, **24**, 675.

MURRAY, N., COPPIN, C. & SWENERTON, K. (1987). Weekly high intensity cisplatin etoposide (HIPE) for far advanced germ cell cancers (GCC). *Proc. ASCO*, **6** (March).

NEWLANDS, E.S., BAGSHAWE, K.D., BEGENT, R.H.J. & 3 others (1986). Current optimum management of anaplastic germ cell tumours of the testis and other sites. *Br. J. Urol.*, **58**, 307.

NICHOLS, C., WILLIAMS, S., TRICOT, G. & 4 others (1988). Phase I study of high dose VP-16 plus carboplatin (CBDCA) with autologous bone marrow rescue (ABMT) in refractory germ cell cancer. *Proc. ASCO*, **7**, 118 (March).

OZOLS, R.F., CORDEN, B.J., JACOB, J., WESLEY, M.N., OSTCHEGA, Y. & YOUNG, R.C. (1984). High-dose cisplatin in hypertonic saline. *Ann. Int. Med.*, **100**, 19.

OZOLS, R.F., IHDE, D.C., LINEHAN, W.M., JACOB, J., OSTCHEGA, Y. & YOUNG, R.C. (1988). A randomized trial of standard chemotherapy *vs.* a high-dose chemotherapy regimen in the treatment of poor prognosis nonseminomatous germ-cell tumors. *J. Clin. Oncol.*, **6**, 1031.

PECKHAM, M.J., BARRETT, A., LIEW, K.H. & 5 others (1983). The treatment of metastatic germ-cell testicular tumours with bleomycin, etoposide and cis-platin (BEP). *Br. J. Cancer*, **47**, 613

PECKHAM, M.J., HORWICH, A., BLACKMORE, C. & HENDRY, W.F. (1985). Etoposide and cis-platin with or without bleomycin as first line chemotherapy for patients with small volume metastases of testicular non-seminoma. *Cancer Treat. Rep.*, **69**, 483.

PECKHAM, M.J., HORWICH, A., EASTON, D. & HENDRY, W.F. (1988). The management of advanced testicular teratoma. *Br. J. Urol.*, **62**, 63.

PERA, M.F., BLASCO, LAFFITA, M.J., MILLS, J. & MONAGHAN, P. (1988). Analysis of cell differentiation lineage in human teratomas using new monoclonal antibodies to cytostructural antigens of embryonal carcinoma cells (in the press).

PIZZOCARO, G., PIVA, L., SALVIONI, R., ZANONI, F. & MILANI, A. (1985). Cisplatin, etoposide, bleomycin first-line therapy and early resection of residual tumour in far-advanced germinal testis cancer. *Cancer*, **56**, 2411.

PRICE, P., HOGAN, S.J. & HORWICH, A. (1988). Tumour proliferation rate as a predictive factor in testicular teratomas. *Br. J. Cancer*, **58**, 525.

ROTH, B.J., GREIST, A., KUBILIS, P.S., WILLIAMS, S.D. & EINHORN, L.H. (1988). Cisplatin-based combination chemotherapy for disseminated germ cell tumors: Long-term follow-up. *J. Clin. Oncol.*, **6**, 1239.

SAMSON, M.K., RIVKIN, S.E., JONES, S.E. & 5 others (1984). Dose-response and dose-survival advantage for high versus low dose cisplatin combined with vinblastine and bleomycin in disseminated testicular cancer. *Cancer*, **53**, 1029.

SLEDGE, G.W., EBLE, J.N., ROTH, B.J. & 3 others (1988). Relation of proliferative activity to survival in patients with advanced germ cell cancer. *Cancer Res.*, **48**, 3964.

STOTER, G., KAYE, S., SLEYFER, D. & 7 others (1986). Preliminary results of BEP and PVB (cisplatin, vinblastine, bleomycin) in high volume metastatic (HVM) testicular non-seminomas. An EORTC-study. *Proc. ASCO*, **5**, 106.

STOTER, G., KAYE, S., JONES, W. & 8 others (1987). Cisplatin (P) and VP16 (E) +/− bleomycin (B) (BEP vs EP) in good risk patients with disseminated non-seminomatous testicular cancer: a randomized EORTC GU Group study. *Proc. ASCO*, **6**, 110 (March).

STOTER, G., SYLVESTER, R., SLEIFER, D.T. & 7 others (1987). Multivariate analysis of prognostic factors in patients with disseminated nonseminomatous testicular cancer: results from a European Organization for research on treatment of cancer multi-institutional Phase III study. *Cancer Res.*, **47**, 2714.

THE MEDICAL RESEARCH COUNCIL WORKING PARTY ON TESTICULAR TUMOURS (1985). Prognostic factors in advanced non-seminomatous germ-cell testicular tumours. Results of a multicentre study. *Lancet*, **i**, 8.

WATSON, J.V., STEWART, J., EVAN, G.I., RITSON, A. & SIKORA, K. (1986). The clinical significance of flow cytometric c-myc oncoprotein quantitation in testicular cancer. *Br. J. Cancer*, **53**, 331.

WETTLAUFER, J.N., FEINER, A.S. & ROBINSON, W.A. (1984). Vincristine, cisplatin and bleomycin with surgery in the management of advanced metastatic nonseminomatous testis tumours. *Cancer*, **53**, 203.

WILLIAMS, S., BIRCH, R., EINHORN, L.H., IRWIN, L., GRECO, F.A. & LOEHRER, P.J. (1987). Treatment of disseminated germ-cell tumours with cisplatin, bleomycin, and either vinblastine or etoposide. *N. Engl. J. Med.*, **316**, 1435.